# Regulation of Anti-Apoptotic SOD2 and BIRC3 in Periodontal Cells and Tissues

**DOI:** 10.3390/ijms22020591

**Published:** 2021-01-08

**Authors:** Birgit Rath-Deschner, Andressa Vilas Boas Nogueira, Svenja Memmert, Marjan Nokhbehsaim, Joni Augusto Cirelli, Sigrun Eick, Nicolai Miosge, Christian Kirschneck, Marco Kesting, James Deschner, Andreas Jäger, Anna Damanaki

**Affiliations:** 1 Department of Orthodontics, Center of Dento-Maxillo-Facial Medicine, University of Bonn, 53111 Bonn, Germany; andreas.jaeger@ukbonn.de; 2 Department of Periodontology and Operative Dentistry, University Medical Center, University of Mainz, 55131 Mainz, Germany; a.nogueira@uni-mainz.de (A.V.B.N.); james.deschner@uni-mainz.de (J.D.); adamanak@uni-mainz.de (A.D.); 3 Department of Orthodontics and Section of Experimental Dento-Maxillo-Facial Medicine, Center of Dento-Maxillo-Facial Medicine, University of Bonn, 53111 Bonn, Germany; svenja.memmert@ukbonn.de; 4 Section of Experimental Dento-Maxillo-Facial Medicine, Center of Dento-Maxillo-Facial Medicine, University of Bonn, 53111 Bonn, Germany; m.saim@uni-bonn.de; 5 Department of Diagnosis and Surgery, School of Dentistry at Araraquara, Sao Paulo State University, UNESP, Araraquara 14801-903, Brazil; joni.cirelli@unesp.br; 6 Department of Periodontology, Laboratory for Oral Microbiology, zmk bern, Zahnmedizinische Kliniken, 53010 Bern, Switzerland; sigrun.eick@zmk.unibe.ch; 7 Department of Prosthodontics, University Medical Center, 37075 Goettingen, Germany; nmiosge@gwdg.de; 8 Department of Orthodontics, University of Regensburg, 93053 Regensburg, Germany; christian.kirschneck@klinik.uni-regensburg.de; 9 Department of Oral and Maxillofacial Surgery, Friedrich-Alexander University Erlangen-Nürnberg (FAU), 91054 Erlangen, Germany; marco.kesting@uk-erlangen.de

**Keywords:** orthodontic tooth movement, periodontitis, gingivitis, *Fusobacterium nucleatum*, periodontal ligament, periodontium

## Abstract

The aim of the study was to clarify whether orthodontic forces and periodontitis interact with respect to the anti-apoptotic molecules superoxide dismutase 2 (SOD2) and baculoviral IAP repeat-containing protein 3 (BIRC3). SOD2, BIRC3, and the apoptotic markers caspases 3 (CASP3) and 9 (CASP9) were analyzed in gingiva from periodontally healthy and periodontitis subjects by real-time PCR and immunohistochemistry. SOD2 and BIRC3 were also studied in gingiva from rats with experimental periodontitis and/or orthodontic tooth movement. Additionally, SOD2 and BIRC3 levels were examined in human periodontal fibroblasts incubated with *Fusobacterium nucleatum* and/or subjected to mechanical forces. Gingiva from periodontitis patients showed significantly higher SOD2, BIRC3, CASP3, and CASP9 levels than periodontally healthy gingiva. SOD2 and BIRC3 expressions were also significantly increased in the gingiva from rats with experimental periodontitis, but the upregulation of both molecules was significantly diminished in the concomitant presence of orthodontic tooth movement. In vitro, SOD2 and BIRC3 levels were significantly increased by *F. nucleatum*, but this stimulatory effect was also significantly inhibited by mechanical forces. Our study suggests that SOD2 and BIRC3 are produced in periodontal infection as a protective mechanism against exaggerated apoptosis. In the concomitant presence of orthodontic forces, this protective anti-apoptotic mechanism may get lost.

## 1. Introduction

The tooth-supporting apparatus, i.e., periodontium, is usually subjected simultaneously to microbial infection and biomechanical forces due to occlusal loading or orthodontic therapy. In the oral cavity, more than 700 different bacterial species have been identified, and some of them have been closely associated with gingivitis and periodontitis, such as *Fusobacterium nucleatum* [1,2,3]. *F. nucleatum* serves as a bridge bacterium between primary and secondary colonizers in dental biofilm formation. Streptococci and Gram-positive rods are the first microorganisms to colonize the pellicle on the tooth surface. These primary colonizers then coaggregate with each other. However, they cannot normally coaggregate with secondary colonizers. *F. nucleatum* is an adhesive bacterium and encodes several adhesins for interspecies interactions. Since *F. nucleatum* can coaggregate with both primary and secondary colonizers, this microorganism is considered a bridge bacterium [4]. It has been reported that many secondary colonizers cannot become part of dental plaque in the absence of *F. nucleatum* [5]. Moreover, coaggregation with *F. nucleatum* seems to be crucial for the survival of anaerobic secondary colonizers in the planktonic state [5,6]. Thus, *F. nucleatum* plays an important role in dental biofilm formation.

Periodontopathogenic bacteria induce inflammatory cells to produce a wide range of proinflammatory mediators, proteases, and osteoclast-activating factors, which can mediate or directly cause destruction of periodontal tissues and structures [7,8]. Animal experiments have provided evidence that occlusal overloading can aggravate periodontal diseases [9,10]. Moreover, in orthodontic therapy, biomechanical forces are used to stimulate and guide remodeling of the periodontium, demonstrating that biomechanical forces can induce soft tissue degradation and hard tissue resorption similar to periodontopathogenic microorganisms [11].

The superoxide dismutase 2 (SOD2) and baculoviral IAP repeat-containing protein 3 (BIRC3), also known as cIAP2, are two molecules that seem to be involved in the actions of microbial and mechanical signals on periodontal cells and tissues [12,13,14,15]. SOD2 can convert toxic superoxide into hydrogen peroxide and diatomic oxygen. By clearing reactive oxygen species, SOD2 protects cells from death and has therefore a well-established anti-apoptotic role in oxidative stress, inflammation, and ionizing radiation [16,17,18]. BIRC3 has modulating effects on inflammation, cell proliferation, and invasion and can also inhibit apoptosis [19,20].

Apoptosis is a form of programmed cell death and plays an important role in development, tissue homeostasis, and wound healing but also in inflammatory diseases, cancer, and other pathologies [21]. Two main signaling pathways of apoptosis, an intrinsic (mitochondria-mediated) and an extrinsic (cell death receptor-mediated) one, have been identified. Furthermore, it was shown that several initiator caspases (CASP), e.g., CASP9, and executioner caspases, e.g., CASP3, mediate the effects of apoptosis. Caspases are inactive zymogens that are activated by cleavage [22,23]. Activation of executioner caspases finally leads to the typical morphological and biochemical characteristics of apoptosis, e.g., cell shrinkage, cell rounding and chromatin condensation, DNA cleavage and fragmentation, nuclear fragmentation, membrane blebbing, apoptotic bodies, and increased protein degradation [21]. It has been suggested that apoptosis may also play a role in the development and progression of periodontitis [24]. For example, several periodontitis-associated risk factors have been shown to exert their negative effects on periodontal cells and tissues via the induction of apoptosis [24,25,26,27]. Furthermore, apoptosis has been shown to be a pathomechanism linking periodontal and systemic diseases [28].

So far, little is known about how and to what extent periodontopathogenic microorganisms and biomechanical loading caused by occlusal forces or orthodontic therapy interact with each other. It was sometimes doubted that biomechanical forces have a significant influence on periodontal diseases in humans, whereas animal and in-vitro experiments, which are better defined and controlled than clinical studies, clearly indicate that mechanical signals can synergize or even counteract the effects of microbial actions [9,10,29,30,31].

In a recently published study, self-reported bruxers had a lower likelihood of periodontitis and better periodontal clinical parameters than non-bruxers, suggesting that frequent mechanical overload does not automatically contribute to periodontal destruction. However, as the investigators acknowledged, the study had some limitations. Single-reporting time self-report of bruxism is not an ideal study approach. In addition, the study was not longitudinal, and no intervention took place, so conclusions regarding causality were not possible [32]. A review that included six articles found that the only effect of bruxism on periodontal structures was an increase in periodontal sensation, while an association with periodontal lesions was absent. The authors therefore concluded that bruxism per se cannot cause periodontal damage. Due to the heterogeneity of the included studies, a meta-analysis could not be performed. Furthermore, a longitudinal evaluation of the temporal relationship and dose-response effects between bruxism and periodontal lesions was lacking, so no conclusions on causality could be drawn [33]. The interesting study was met with criticism but highlights the need for future studies on the interaction between mechanical forces and periodontal infection [34].

The present in-vitro and in-vivo study was therefore designed to clarify whether orthodontic forces and periodontitis interact with respect to the regulation of the two anti-apoptotic molecules SOD2 and BIRC3.

## 2. Results

### 2.1. Regulation of SOD2 and BIRC3 by Periodontal Infection in Human Gingiva

**Figure 1:**
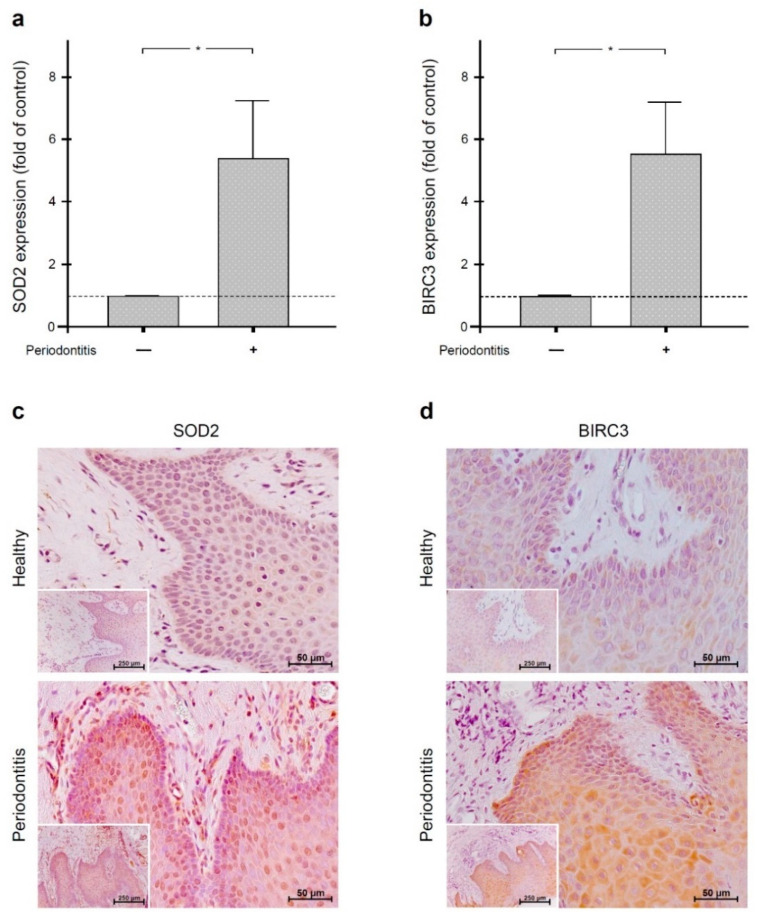
SOD2 (**a**) and BIRC3 (**b**) gene expressions in the gingiva of periodontally healthy (*n* = 7) and periodontitis (*n* = 7) subjects. Bars show mean ± SEM. * significant (*p* < 0.05) difference between gingiva of periodontally healthy and periodontitis subjects. SOD2 (**c**) and BIRC3 (**d**) protein in gingival biopsies from periodontally healthy and periodontitis subjects. Representative histological sections are shown.

First, we analyzed the SOD2 and BIRC3 gene expressions in gingiva of periodontally healthy and periodontitis subjects. The quantitative real-time PCR analysis revealed significantly (*p* < 0.05) increased SOD2 and BIRC3 levels in gingival biopsies of periodontitis patients as compared to gingival samples from periodontally healthy individuals (Figure 1a,b). Then, gingival biopsies from periodontally healthy and periodontitis subjects were also studied for the presence of SOD2 and BIRC3 by immunohistochemistry. Although these molecules were detectable in the gingiva of both periodontally healthy and diseased individuals, biopsies from periodontitis patients showed more pronounced immunostaining for SOD2 and BIRC3, especially in the epithelial tissue, thereby confirming our transcriptional results for these molecules (Figure 1c,d).

### 2.2. Regulation of SOD2 and BIRC3 by Periodontal Infection and/or Orthodontic Tooth Movement in Rat Gingiva

**Figure 2:**
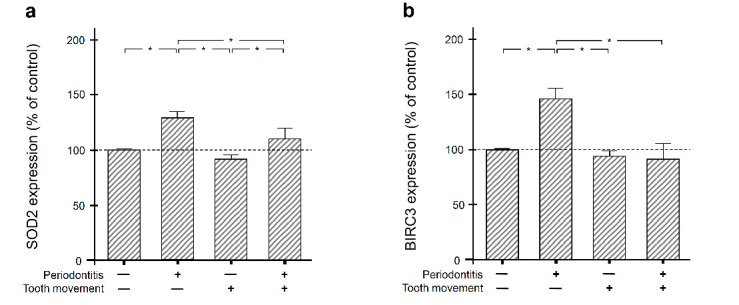
SOD2 (**a**) and BIRC3 (**b**) gene expressions in gingiva of rats with experimental periodontitis and/or orthodontic tooth movement. Animals, which were not subjected to experimental periodontitis and/or orthodontic tooth movement, served as control. *n* = 4 animals/group. Bars show mean ± SEM. * significant (*p* < 0.05) difference between animal groups.

Next, we sought to clarify whether the actions of periodontal infection on gingival SOD2 and BIRC3 are modulated by orthodontic forces. Therefore, rats were subjected to experimental periodontitis and/or orthodontic tooth movement. As evidenced by real-time PCR, the transcriptional levels of SOD2 and BIRC3 were significantly (*p* < 0.05) elevated at gingival sites of experimental periodontitis (Figure 2a,b). However, when these sites were also affected by experimental orthodontic tooth movement, the periodontitis-induced upregulation of SOD2 and BIRC3 was significantly (*p* < 0.05) reduced again (Figure 2a,b), demonstrating an inhibitory effect of orthodontic forces on these molecules in periodontal infection.

### 2.3. Regulation of SOD2 and BIRC3 by F. nucleatum and/or Biomechanical Forces in Human Periodontal Fibroblasts

**Figure 3:**
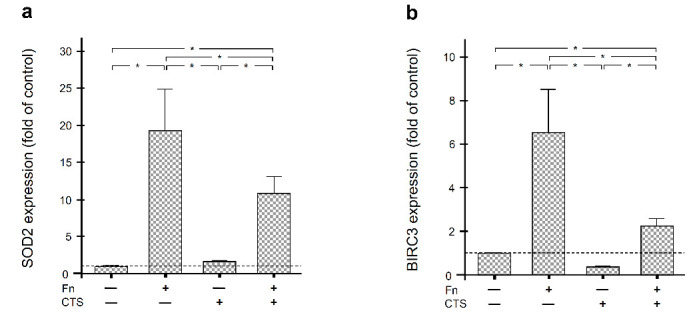
SOD2 (**a**) and BIRC3 (**b**) gene expressions in human periodontal fibroblasts. Human periodontal fibroblasts were subjected to *F. nucleatum* stimulation and/or constant tensile strain (CTS) for 1 d. Untreated fibroblasts served as control. *n* = 9/group. Bars show mean ± SEM. * significant (*p* < 0.05) difference between groups.

In order to study whether the interaction of bacterial and mechanical insults can also be observed in humans, periodontal fibroblasts were exposed to *F. nucleatum* and/or constant tensile strain (CTS) for 1 d. The periodontopathogen *F. nucleatum* caused a strong and significant (*p* < 0.05) upregulation of SOD2 and BIRC3, as shown in Figure 3a,b. Like in rats, mechanical forces significantly (*p* < 0.05) counteracted the stimulatory effect of the bacteria on these genes (Figure 3a,b).

### 2.4. Increased Gingival Levels of Apoptotic Markers in Periodontal Infection

**Figure 4:**
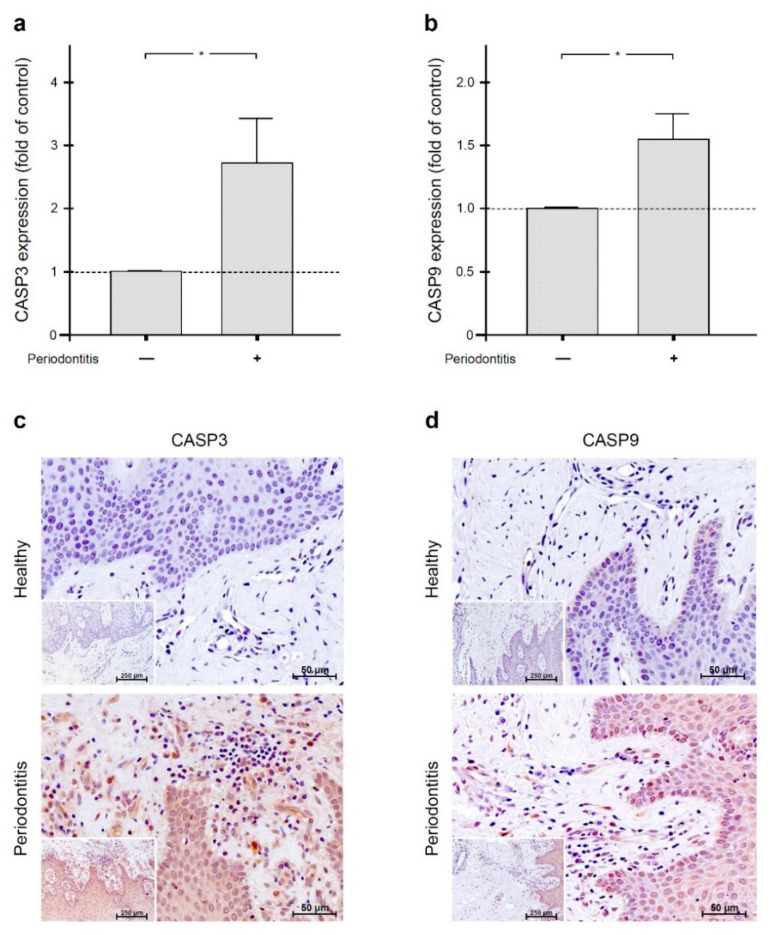
CASP3 (**a**) and CASP9 (**b**) gene expressions in the gingiva of periodontally healthy (*n* = 7) and periodontitis (*n* = 7) subjects. Bars show mean ± SEM. * significant (*p* < 0.05) difference between gingiva of periodontally healthy and periodontitis subjects. Active CASP3 (**c**) and CASP9 (**d**) protein in gingival biopsies from periodontally healthy and periodontitis subjects. Representative histological sections are shown.

Since SOD2 and BIRC3 can inhibit apoptosis, we additionally investigated the levels of the apoptotic markers CASP3 and CASP9 in gingival biopsies from periodontally healthy and periodontitis subjects. As analyzed by quantitative real-time PCR, both CASP3 and CASP9 were significantly (*p* < 0.05) increased in gingival samples from periodontitis patients as compared to gingival biopsies from periodontally healthy individuals (Figure 4a,b). These transcriptional results were further confirmed at the protein level by immunohistochemistry, i.e., more pronounced immunostaining for active CASP3 and CASP9 was found in gingiva from periodontitis patients in comparison to periodontally healthy gingiva. In inflamed gingiva, active CASP3 and CASP9 were detected mainly in gingival epithelial cells, gingival fibroblasts, and cells of the inflammatory infiltrate, such as neutrophils and lymphocytes (Figure 4c,d).

### 2.5. Anti-Apoptotic Effect of SOD2 and BIRC3 on Human Periodontal Fibroblasts

**Figure 5:**
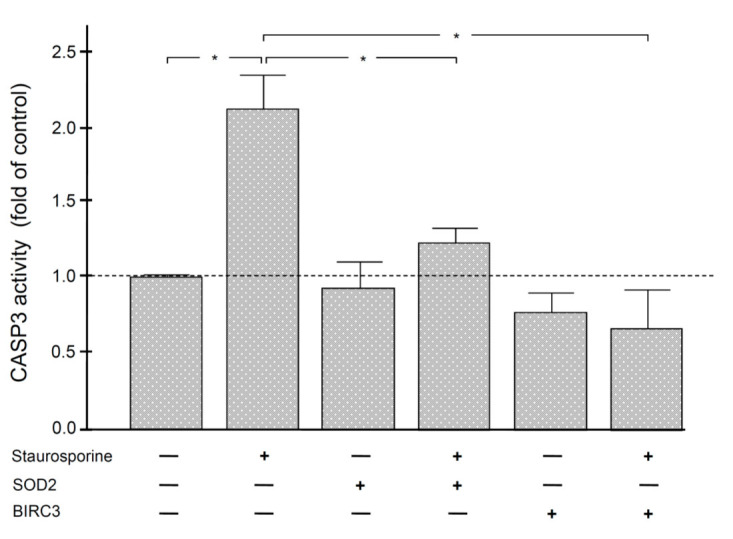
CASP3 activity in human periodontal fibroblasts. Cells were incubated with staurosporine, SOD2, BIRC3, or the combination of staurosporine with SOD2 or BIRC3 for 1 d. Untreated fibroblasts served as control. *n* = 5/group. Bars show mean ± SEM. * significant (*p* < 0.05) difference between groups.

Finally, we analyzed by fluorescence whether SOD2 and BIRC3 can exert anti-apoptotic effects on periodontal fibroblasts. As expected, staurosporine, an established inducer of apoptosis, resulted in elevated activity of CASP3 in these structural periodontal cells. However, the staurosporine-stimulated CASP3 activity could be significantly inhibited by both SOD2 and BIRC3 (Figure 5).

## 3. Discussion

**Figure 6:**
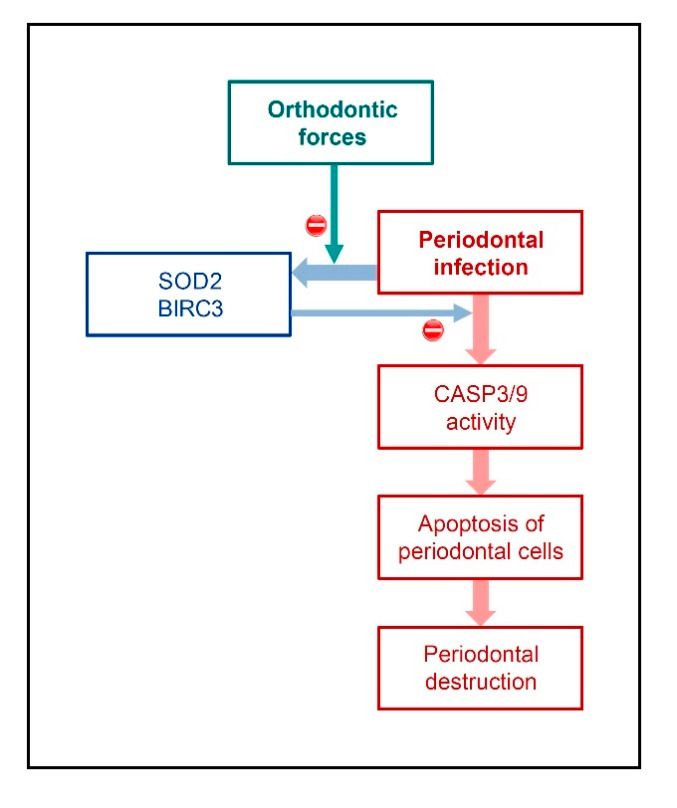
Hypothesis on the modulation of periodontal infection by orthodontic forces. Periodontal infection leads to increased activity of CASP3 and CASP9 in periodontal tissues, which promotes apoptosis of periodontal cells, contributing to periodontal destruction. Periodontal infection also leads to the upregulation of SOD2 and BIRC3, which can inhibit CASP3/9 activity, providing a protective mechanism against apoptosis and tissue damage. However, orthodontic forces could counteract the bacteria-induced upregulation of SOD2 and BIRC3, which would then lead to less inhibition of CASP3/9 activity, more apoptosis, and thus increased periodontal destruction.

Our in-vitro and in-vivo study revealed that the anti-apoptotic molecules SOD2 and BIRC3 are upregulated in a microbial environment and that the bacteria-induced upregulation of these molecules is inhibited by mechanical forces. Moreover, our transcriptional and protein analyses showed elevated levels of the apoptotic markers CASP3 and CASP9 in gingiva from periodontitis patients in comparison to periodontally healthy gingiva, indicating increased apoptotic activity at sites of periodontitis. In addition, our in-vitro experiments demonstrated that the staurosporine-stimulated CASP3 activity in human periodontal fibroblasts could be inhibited by both SOD2 and BIRC3, providing original evidence for the anti-apoptotic effects of SOD2 and BIRC3 on periodontal structural cells. Therefore, our data suggest that SOD2 and BIRC3 are produced in periodontal infection as a possible protective mechanism against apoptosis and, thereby, tissue damage. However, in the concomitant presence of periodontal infection and orthodontic forces, this protective anti-apoptotic mechanism may get lost, which could result in an aggravated destruction of periodontal tissues and structures (Figure 6).

SOD2 converts toxic superoxide into hydrogen peroxide and diatomic oxygen, thereby protecting cells from death. Besides its well-established anti-apoptotic role in oxidative stress, SOD2 is involved in inflammation and reaction to ionizing radiation [16,17,18]. Like SOD2, BIRC3 can also inhibit apoptosis [19,20]. Our investigation revealed higher SOD2 and BIRC3 gene expression and protein levels in gingiva from periodontitis patients as compared to periodontally healthy gingiva, suggesting a critical role of these molecules in periodontal infection and inflammation. Since these molecules have anti-apoptotic effects, the observed SOD2 and BIRC3 upregulation may represent a protective mechanism against the loss of periodontal structural cells and tissues. However, if inflammatory cells are protected against apoptosis, the bacteria-induced inflammation may become chronic and exaggerated, which would promote periodontal destruction. Therefore, future studies should further clarify the anti-apoptotic actions of SOD2 and BIRC3 on periodontal structural and inflammatory cells.

Some previous studies have also focused on the presence and role of SOD2 in periodontal tissues. For example, it has been reported that *F. nucleatum* and *Porphyromonas gingivalis* can regulate SOD2 expression in oral epithelial cells [35,36,37]. Similarly, *P. gingivalis* has been shown to induce the expression of SOD2 in human gingival fibroblasts [12]. Moreover, gingival biopsies from human subjects with experimental gingivitis demonstrated an increased SOD2 expression, which declined upon resolution of the gingival inflammation [38]. In addition, a SOD2 increase has also been found in gingival tissues from periodontitis patients [13,39]. Furthermore, *F. nucleatum* has been shown to stimulate SOD2 in neutrophils from healthy donors [40]. These findings support our observation that SOD2 is increased in periodontally diseased gingiva.

So far, little is known about the role of BIRC3 in oral physiology and pathophysiology. BIRC3 was recently identified as one of many other regulated genes in *F. nucleatum*-stimulated rat osteoblasts by whole-transcriptome analyses [14]. Moreover, periodontal ligament (PDL) cells have been shown to produce BIRC3, which could be increased by high tensile forces [15,41]. These studies concur with our findings in that BIRC3 is regulated by microbial and mechanical signals in osteoblast- and fibroblast-like cells. Interestingly, gingival epithelial cells infected with *P. gingivalis* have not shown elevated levels of BIRC3 [42]. However, this lack of change in BIRC3 levels seems to be less due to the epithelial cell type than to the inability of *P. gingivalis* to stimulate BIRC3 synthesis, as our immunohistochemical analysis also showed increased BIRC3 protein levels in the inflamed gingival epithelium. Interestingly, BIRC3 may also play a role in the association between periodontitis and atherosclerosis because BIRC3 was down-regulated in aortic tissues after oral polybacterial infection with *P. gingivalis*, *Treponema denticola*, *Tannerella forsythia*, and *F. nucleatum* in ApoE null mice [43].

In our study, the upregulation of SOD2 and BIRC3 in human gingiva due to periodontal infection and thereby inflammation was confirmed using an animal model with experimental periodontitis and an in-vitro study on periodontal fibroblasts. These pre-clinical experiments also provided original evidence that the stimulatory effects of periodontal bacteria on SOD2 and BIRC3 are counteracted by mechanical forces. This observation suggests that the protective anti-apoptotic actions of SOD2 and BIRC3 in periodontal infection may get lost when periodontal cells and tissues are concomitantly subjected to orthodontic forces. It is possible that the additional presence of mechanical overload overwhelms the ability of the periodontium to respond to microbial stress to some degree. Adaptation of the periodontium thus seems possible only to a certain degree, as in any living system. Future studies should be dedicated to exploring the underlying mechanisms by which orthodontic forces inhibit the bacteria-induced upregulation of SOD2 and BIRC3.

Increased expression, protein, and activity levels of CASP3 and CASP9 have been found in gingival samples from sites of periodontitis as compared to healthy sites [44,45,46,47], which is in accordance with our study, which demonstrated increased CASP3 and CASP9 levels at transcriptional and protein level in gingiva from periodontitis patients. These data suggest that apoptosis is implicated in periodontal inflammation and destruction. Additionally, elevated gingival crevicular fluid and serum concentrations of CASP3 and CASP9 have been observed in periodontal disease, i.e., gingival inflammation [48,49,50]. Moreover, *P. gingivalis* has been reported to decrease CASP3 and CASP9 expressions in gingival epithelial cells but increase the expression of these molecules in gingival fibroblasts [44]. Furthermore, *Filifactor alocis* has been shown to induce apoptosis in gingival epithelial cells through pathways that involved CASP3 but not CASP9 [51]. In normal and diabetic rats, experimental periodontitis with *Aggregatibacter actinomycetemcomitans* could enhance apoptosis through a CASP3-dependent mechanism [52]. In the present study, the effect of mechanical forces and their interactions with periodontal bacteria on CASP3 and CASP9 was not investigated. Some studies have shown that mechanical forces can induce apoptosis and activation of CASP3 and CASP9 in human periodontal cells [53,54,55]. Future studies should therefore also focus on the regulation of CASP3 and CASP9 by mechanical forces in the presence and absence of periodontal infection.

Although SOD2 and BIRC3 are known to have generally anti-apoptotic effects, we wanted to know if they also have anti-apoptotic effects on periodontal fibroblasts. As expected, staurosporine, an established inducer of apoptosis, resulted in elevated activity of CASP3 in these structural periodontal cells. However, the staurosporine-stimulated CASP3 activity could be significantly inhibited by both SOD2 and BIRC3, which was shown for the first time and underlines the special role of these two molecules in the periodontium. Since both the intrinsic and the extrinsic pathway lead to the cleavage and activation of the executioner CASP3 and thus to DNA fragmentation, we used CASP3 as the outcome variable [21,24,56].

Human gingival biopsies from periodontally healthy and diseased individuals could be easily obtained for the SOD2 and BIRC3 analyses. However, in order to study the interactions of periodontal infection and orthodontic forces in humans, gingival biopsies from sites of periodontitis together with orthodontic treatment would be required. Since the availability of such biopsies is very restricted, an animal model was chosen to investigate the interactions of periodontal infection and orthodontic tooth movement. In the present study, gingival SOD2 and BIRC3 protein levels were determined by immunohistochemistry but not by enzyme-linked immunosorbent assay, which should be performed in future studies to confirm our data.

Human periodontal fibroblasts were used in our in-vitro experiments. Interestingly, our immunohistochemical analyses revealed pronounced SOD2 and BIRC3 levels in gingival epithelium. Further studies should therefore investigate the regulation of these two molecules by bacteria and/or mechanical forces also in gingival epithelial cells.

In our experiments, periodontal fibroblasts were treated with *F. nucleatum*, which is a Gram-negative, anaerobic, invasive periodontopathogen with a well-established and critical role in biofilm formation and, therefore, gingivitis and periodontitis [2,3]. In previous studies, we compared the effects of *F. nucleatum* and the keystone pathogen *P. gingivalis* on periodontal cells and found that the effects of both pathogens were very similar [57,58,59]. However, whether *P. gingivalis* actually causes similar effects on SOD2 and BIRC3 as *F. nucleatum* should be clarified in further studies. Furthermore, the fibroblasts were treated with a suspension of *F. nucleatum*, which was subjected to intensive ultrasonication before application. This suspension mainly contained disrupted cell wall particles including a high amount of lipopolysaccharide. Future studies should also focus on the effects of live *F. nucleatum* on SOD2 and BIRC3 in periodontal cells and tissues. Interestingly, the effect of *F. nucleatum* on the SOD2 and BIRC3 levels was similar to the actions of clinical periodontal infection, confirming the important etiological role of *F. nucleatum* in periodontitis. However, periodontitis is caused by a complex multispecies biofilm [8]. Future in-vitro studies on the interactions between microbial and mechanical signals should also include living complex biofilms.

## 4. Materials and Methods

### 4.1. Culture and Treatment of Human Periodontal Fibroblasts

**Figure 7:**
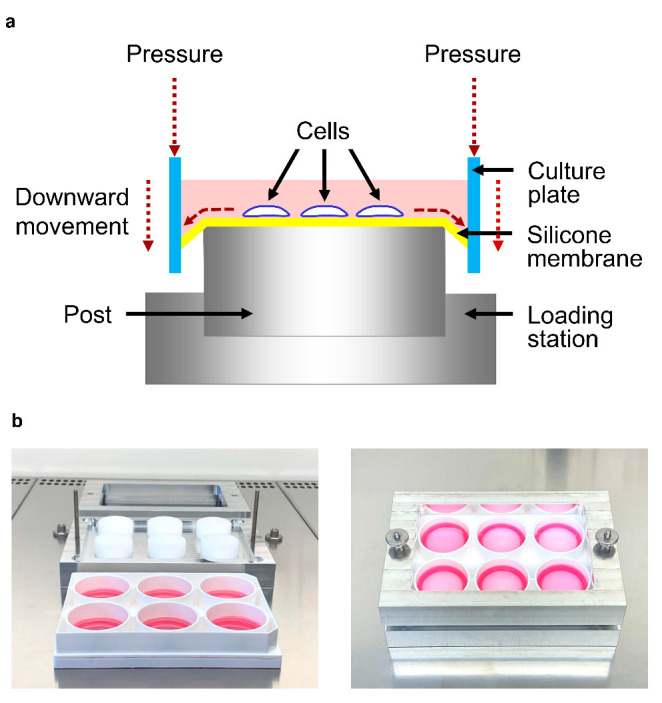
Schematic view of the cell stretch device (**a**). The lower part of the device (loading station) was equipped with cylindrical posts. The BioFlex culture plate was positioned in such a way that the posts were centered beneath the flexible-bottom wells (silicone membranes) of the plate. Tightening the upper part of the device to the lower part by two screws caused a downward movement of the upper part, which resulted in stretching the silicone membranes and thereby the cells attached to them. Dissembled (left) and assembled (right) stretch device with a BioFlex culture plate for the application of static tensile forces to cells (**b**).

Human periodontal ligament fibroblasts were harvested from caries-free and periodontally healthy teeth, which were removed during wisdom tooth surgery or which had to be extracted for orthodontic reasons in the Department of Oral Surgery, University of Bonn. Approval of the Ethics Committee of the University of Bonn and written informed consent from the patients or their parents were obtained (#043/11). The fibroblasts were derived from the middle part of the root surface, grown in Dulbecco’s minimal essential medium (DMEM, Invitrogen, Karlsruhe, Germany) supplemented with 10% fetal bovine serum (FBS, Invitrogen), 100 U/mL penicillin, and 100 μg/mL streptomycin (Invitrogen) at 37 °C in a humidified atmosphere of 5% CO_2_ and used between passages 3 and 5. Before the experiments, the FBS concentration was reduced to 1%. Fibroblasts were treated with *F. nucleatum* ATCC 25586 (OD_660_: 0.05) for 1 d. The microorganisms had been suspended in phosphate-buffered saline (OD_660_ = 1.0, equivalent to 1.2 × 10^9^ bacterial cells/mL) and subjected twice to ultra-sonication (160 W for 15 min) before the experiments. Fibroblasts were also subjected to constant tensile strain (CTS) with a cell strain device for 1 d, as in our previous studies (Figure 7a,b) [60,61,62]. In addition, fibroblasts were exposed simultaneously to *F. nucleatum* and CTS. Untreated fibroblasts served as controls.

### 4.2. Human Gingival Biopsies from Periodontally Healthy and Diseased Subjects

Gingival tissue samples were obtained from periodontally healthy sites of seven periodontitis-free subjects and from inflamed sites of seven periodontitis patients. Only one gingival biopsy from each subject was used for the study. Periodontally healthy sites were characterized by the following parameters: gingival index = 0 (no clinical inflammation), periodontal pocket depths ≤ 3 mm as well as no clinical attachment and radiographic bone loss. Periodontitis sites had a gingival index ≥ 1 (clinical inflammation), periodontal pocket depths ≥ 5 mm, as well as clinical attachment and radiographic bone loss. Healthy gingiva samples became available during wisdom tooth surgery in the Department of Oral Surgery, University of Bonn. Inflamed gingival biopsies originated from periodontitis patients whose teeth had to be extracted for periodontal reasons. Presence of smoking and/or systemic diseases were exclusion criteria. Approval of the Ethics Committee of the University of Bonn and written informed consent from the patients or their parents were obtained (#043/11). Gingival tissues were either stored immediately in RNA stabilization reagent (RNAlater) (Qiagen, Hilden, Germany) and kept in the −80 °C freezer until use or fixed in formaldehyde for histological examination [60,61,63].

### 4.3. Rat Gingival Biopsies and Animal Model

In order to study the regulation of microbial actions by orthodontic forces in vivo, an animal model was applied [60,63]. The in-vivo experiments were carried out according to the ARRIVE (Animal Research: Reporting of In-Vivo Experiments) guidelines. Approval from the Ethical Committee on Animal Experimentation at the School of Dentistry at Araraquara, São Paulo State University—UNESP (Protocol Number: 23/2012) was obtained. Standard laboratory food and water ad libitum were provided to the adult 300 g Holtzman rats (*n* = 32), which were housed in an animal facility. As previously published, experimental periodontitis was induced with cotton ligatures tied around the cervical area of the first molars in the upper jaw. The knot was placed mesially under anesthesia with intramuscular injections of ketamine chlorhydrate 10% (0.08 mL/100 g body weight) and xylazine chlorhydrate 2% (0.04 mL/100 g body weight). Four experimental groups were established: (1) control, (2) experimental periodontitis, (3) orthodontic tooth movement, and (4) combination of experimental periodontitis and orthodontic tooth movement. For the orthodontic tooth movement, a closed coil nickel-titanium spring (Sentalloy^®^, GAC, Dentsply) providing a relatively constant force of 25 g was connected between the first molar and maxillary central incisor teeth of the upper jaw. Displacement of the 0.20 mm steel wire (CrNi, 55.01.208, Morelli, Brazil) was prevented by the preparation of grooves on the maxillary central incisor teeth and the subsequent coverage of the wire in the groove with a thin layer of composite resin. Placement of the spring on the maxillary first molars was also accomplished with composite resin. In order to prevent occlusal interference, the first molars of the lower jaw were removed. For analysis, four rats from each experimental group were sacrificed at 6 d. Afterwards, the gingiva of the first molars of the upper jaw was gently harvested for RNA extraction and subsequent real-time PCR.

### 4.4. Analyses of Gene Expressions by Quantitative Real-Time Polymerase Chain Reaction (PCR)

Total RNA was extracted with the RNeasy Mini Kit (Qiagen) according to the manufacturer’s protocol, and analysis of RNA concentration was performed with a NanoDrop ND-2000 (Thermo Fisher Scientific, Wilmington, DE, USA) spectrophotometer. Four to 500 ng of total RNA was used for reverse transcription into cDNA with the iScript™ Select cDNA Synthesis Kit (Bio-Rad Laboratories, Munich, Germany) at 42 °C for 90 min followed by 85 °C for 5 min according to the manufacturer’s instruction. The SOD2, BIRC3, CASP3, and CASP9 gene expressions were subsequently analyzed in triplicate with QuantiTect Primers (Qiagen), SYBR Green QPCR Master Mix (Bio-Rad), and the iCycler iQ™ Real-Time PCR Detection System (Bio-Rad). The amplification comprised an initial denaturation at 95 °C for 5 min, followed by 40 cycles of denaturation at 95 °C for 10 s and a combined annealing/extension at 60 °C for 30 s. Glyceraldehyde-3-phosphate dehydrogenase (GAPDH) served as a housekeeping gene.

### 4.5. Protein Analysis by Immunohistochemistry

Human gingiva was fixed in 4% formaldehyde (Merck, Darmstadt, Germany) for 2 d. After hydration and dehydration in an ascending ethanol series (AppliChem, Darmstadt, Germany), embedding in paraffin (McCormick Scientific, Richmond, IL, USA), sectioning at 2.5 µm thickness, and mounting on glass slides (Engelbrecht, Edermünde, Germany), the samples were dried at 37 °C overnight. Afterwards, deparaffinization and rehydration were performed, and the endogenous peroxidase was blocked with 0.3% methanol (Merck)/30% H_2_O_2_ (Merck) solution for 10 min. Sections for cleaved CASP3 detection were pretreated with EDTA-Buffer at 80 °C for 20 min followed by a cooling-down period of 15 min at room temperature. Next, sections were incubated with rabbit anti-SOD2 (Abcam, Berlin, Germany; 1:100), anti-BIRC3 (Abcam; 1:100), anti-cleaved CASP3 (Cell Signaling Technology, Frankfurt, Germany; 1:25), or anti-cleaved CASP9 (Invitrogen; 1:100) polyclonal antibodies in a humid chamber at 4 °C overnight (anti-SOD2, anti-BIRC3, and anti-cleaved CASP3) or room temperature for 1 h (anti-cleaved CASP9). Sections were rinsed and incubated with goat anti-rabbit IgG-HRP secondary antibody (Dako, Hamburg, Germany) at room temperature for 30 min. Peroxidase activity was visualized with a 3,3′-diaminobenzidine chromogen (Thermo Fisher Scientific, Dreieich, Germany). Finally, slides were rinsed and counterstained with Mayer’s hematoxylin (Merck) for 30 s, dehydrated, and cover-slipped for light microscopy. Pictures were taken and analyzed with the Axioskop 2 microscope and AxioVision 4.7 software (Carl Zeiss, Jena, Germany).

### 4.6. Measurement of CASP3 Activity

Caspase activity was quantified using the fluorometric kit Caspase 3 Multiplex Activity Assay (ab219915, Abcam) according to the manufacturer’s protocol. Cells from the PDL cell line PDL26 were grown in a 96-well plate at 2 × 10^4^ cells per well. The cell line was obtained from the third molar of a healthy 26-year-old male non-smoker after written consent according to the ethics regulations of the University of Goettingen (file no.: 27/2/09). Immortalization with human Telomerase Reverse Transcriptase has been described before [64]. The cells were treated with 10 nM staurosporine (Sigma-Aldrich, Munich, Germany), 60 ng/mL SOD2 (Abcam), 60 ng/mL BIRC3 (Abcam), or staurosporine in combination with SOD2 or BIRC3 for 1 d. Untreated cells served as control. Then, 100 μL/well of caspase assay loading solution was added to each well, and the plate was incubated at room temperature for 60 min and protected from light. Subsequently, the fluorescence intensity was measured using a SpectraMax iD5 Multi-Mode microplate reader (Molecular Devices, CA, USA) at specific wavelengths (Ex/Em = 535/620 nm). For data analysis, the blank readings were subtracted from all measurements, and the CASP3 activity of each group was determined in relation to the control group.

### 4.7. Data Analysis

Calculation of mean values and standard errors of the mean as well as testing for significant differences between groups was performed with the IBM SPSS Statistics software (Version 22, IBM SPSS, Chicago, IL, USA). Statistically significant (*p* < 0.05) differences between the groups were identified by t-test, Mann-Whitney-U test, or ANOVA followed by the post-hoc Tukey’s or Dunnett’s multiple comparison test.

## 5. Conclusions

Our in-vitro and in-vivo data show that the anti-apoptotic molecules SOD2 and BIRC3 are upregulated in a microbial environment and that the bacteria-induced upregulation of both molecules is diminished by experimental orthodontic forces. Hence, these results suggest that SOD2 and BIRC3 are produced in periodontal infection as a protective mechanism against apoptosis and, therefore, tissue damage. However, in the concomitant presence of periodontal infection and orthodontic forces, this protective mechanism may get lost, which could then lead to aggravated periodontal destruction.

## Data Availability

Data sharing is not applicable to this article.

## Abbreviations

ATCC	American Type Culture Collection
BIRC3	baculoviral IAP repeat-containing protein 3
CASP	caspase
cDNA	complementary desoxyribonucleic acid
CTS	constant tensile strain
DMEM	Dulbecco’s modified Eagle’s medium
DNA	desoxyribonucleic acid
EDTA	ethylenediaminetetraacetic acid
FBS	fetal bovine serum
GAPDH	glyceraldehyde-3-phosphate dehydrogenase
HRP	horseradish peroxidase
OD	optical density
PCR	polymerase chain reaction
PDL	periodontal ligament
RNA	ribonucleic acid
SOD2	superoxide dismutase 2
